# Reverse Methanogenesis and Respiration in Methanotrophic Archaea

**DOI:** 10.1155/2017/1654237

**Published:** 2017-01-05

**Authors:** Peer H. A. Timmers, Cornelia U. Welte, Jasper J. Koehorst, Caroline M. Plugge, Mike S. M. Jetten, Alfons J. M. Stams

**Affiliations:** ^1^Laboratory of Microbiology, Wageningen University, Stippeneng 4, 6708 WE Wageningen, Netherlands; ^2^Wetsus, European Centre of Excellence for Sustainable Water Technology, Oostergoweg 9, 8911 MA Leeuwarden, Netherlands; ^3^Soehngen Institute of Anaerobic Microbiology, Heyendaalseweg 135, 6525 AJ Nijmegen, Netherlands; ^4^Department of Microbiology, Radboud University, Heyendaalseweg 135, 6525 AJ Nijmegen, Netherlands; ^5^Laboratory of Systems and Synthetic Biology, Wageningen University, Stippeneng 4, 6708 WE Wageningen, Netherlands; ^6^TU Delft Biotechnology, Julianalaan 67, 2628 BC Delft, Netherlands; ^7^Centre of Biological Engineering, University of Minho, Campus de Gualtar, 4710-057 Braga, Portugal

## Abstract

Anaerobic oxidation of methane (AOM) is catalyzed by anaerobic methane-oxidizing archaea (ANME) via a reverse and modified methanogenesis pathway. Methanogens can also reverse the methanogenesis pathway to oxidize methane, but only during net methane production (i.e., “trace methane oxidation”). In turn, ANME can produce methane, but only during net methane oxidation (i.e., enzymatic back flux). Net AOM is exergonic when coupled to an external electron acceptor such as sulfate (ANME-1, ANME-2abc, and ANME-3), nitrate (ANME-2d), or metal (oxides). In this review, the reversibility of the methanogenesis pathway and essential differences between ANME and methanogens are described by combining published information with domain based (meta)genome comparison of archaeal methanotrophs and selected archaea. These differences include abundances and special structure of methyl coenzyme M reductase and of multiheme cytochromes and the presence of menaquinones or methanophenazines. ANME-2a and ANME-2d can use electron acceptors other than sulfate or nitrate for AOM, respectively. Environmental studies suggest that ANME-2d are also involved in sulfate-dependent AOM. ANME-1 seem to use a different mechanism for disposal of electrons and possibly are less versatile in electron acceptors use than ANME-2. Future research will shed light on the molecular basis of reversal of the methanogenic pathway and electron transfer in different ANME types.

## 1. Introduction 

### 1.1. Anaerobic Methane-Oxidizing Archaea (ANME)

**Figure 1:**
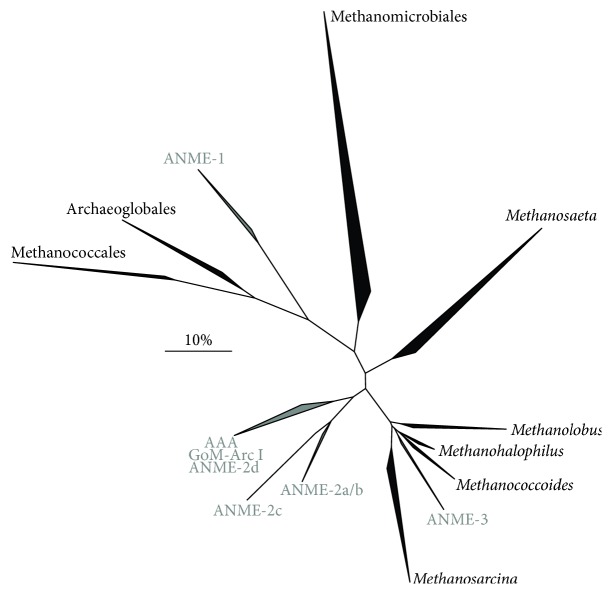
Phylogenetic tree of full length archaeal 16S rRNA sequences showing all methanotrophic clades so far described (grey) and other archaeal clades used in our domain based (meta)genome comparison (black). The tree was constructed with the ARB software package (version arb-6.0.1.rev12565) [49] using 2800 sequences from the SILVA SSURef NR 99 database (release 119.1) [50]. Trees were calculated by maximum likelihood analysis (RAxML, PHYML) and the ARB neighbor-joining method with terminal filtering and the Jukes-Cantor correction. Resulting trees were compared manually and a consensus tree was constructed. Sulfolobales as outgroup was removed after tree calculations. The scale bar represents the percentage of changes per nucleotide position.

Anaerobic methane-oxidizing archaea (ANME) perform anaerobic oxidation of methane (AOM) via reversal of the methanogenic pathway. ANME were first discovered in marine sediments where AOM was coupled to sulfate reduction (SR) (Table 1, reaction (1)). Here, ANME formed metabolically interdependent consortia with sulfate-reducing bacteria (SRB) that belong to the Deltaproteobacteria [1–3]. Three distinct methanotrophic groups were identified: ANME-1 (subclusters a and b), ANME-2 (subclusters a, b, and c), and ANME-3. The ANME-1 cluster is related to Methanomicrobiales and Methanosarcinales but forms a separate cluster [2], ANME-2 are related to cultivated members of the Methanosarcinales [4], and ANME-3 are more related to* Methanococcoides* spp. [5] (Figure 1). The ANME clades are not monophyletic with each other and the phylogenetic distance between the subgroups is large, with 16S rRNA gene sequence similarity of only 75–92% [6]. Subclusters ANME-2a and ANME-2b form a coherent clade that is distinguished from ANME-2c [7] and are therefore often grouped together as ANME-2a/b (Figure 1). The wide phylogenetic distribution is reflected in the ecological niche adaptation of the different ANME clades. ANME clades involved in sulfate-dependent AOM (S-AOM) co-occur in many different marine environments, except for ANME-3 that was mainly found in mud volcanoes and in some seep sediments [6, 8, 9]. In marine sediments, a distinct zonation occurs where ANME-2a/b dominate upper layers and ANME-2c and/or ANME-1 abundance increases in deeper zones, indicating ecological niche separation [10–15]. ANME also form a versatile partnership with non-SRB such as beta-proteobacteria [16] and Verrucomicrobia [17]. ANME, and especially ANME-1, have also been observed without a (closely associated) bacterial partner [5, 12, 13, 18–22]. It was therefore suggested that ANME could perform AOM alone, using electron acceptors such as metal oxides, or perform other processes such as methanogenesis [23, 24]. Indications exist that AOM can be coupled to the reduction of different metal (oxides) (Table 1, reactions (3)–(5)), but limited experimental evidence exists to date that ANME are responsible for this reaction (discussed in Section 3.3). Besides marine environments, ANME involved in S-AOM can be found in terrestrial [25, 26] and freshwater ecosystems [27].

A member of a fourth subcluster, “*Candidatus (Ca.)* Methanoperedens nitroreducens,” was recently discovered to perform nitrate-dependent AOM (N-AOM) [28] (Table 1, reaction (2)). This cluster was named “ANME-2d” [29] but later renamed to “GOM Arc I” [30] and “AOM-associated archaea (AAA)” [6]. Phylogenetic analysis shows that the ANME-2d cluster is monophyletic with “*Ca.* M. nitroreducens*”* and other AAA/GoM Arc I sequences, but distinct from other ANME-2 subclusters (Figure 1). ANME-2d were initially enriched in bioreactors inoculated with freshwater samples [28, 31–33]. As ANME-2d archaea were only recently recognized, their environmental preferences remain insufficiently studied. So far they have been found in freshwater canals [31], soils and rice paddy fields [34–36], lakes and rivers [35], and wastewater treatment plants [33]. In situ AOM activity of ANME-2d was determined recently for the first time [36]. More ANME phylotypes in different environments and possibly new archaeal clades involved in AOM may yet have to be discovered. For example, methyl coenzyme M reductase A genes (*mcrA*) from Bathyarchaeota (formerly known as Miscellaneous Crenarchaeota Group) and from the new archaeal phylum Verstraetearchaeota were recently found, indicating their involvement in methane metabolism [37, 38].

This review focusses on archaea performing AOM through the reversal of the methanogenesis pathway. We describe the reversibility of the central methanogenic pathway, including the key enzyme in methanogenesis and anaerobic methanotrophy (i.e., methyl coenzyme M reductase, Mcr). The possibility of methanogens to perform methane oxidation and of ANME to perform methanogenesis is also addressed. Lastly, the physiological adaptations of ANME to perform respiration using different electron acceptors during AOM are discussed.

### 1.2. ANME versus Methanogens: Domain Based (Meta)Genome Comparison

In order to find additional differences between archaeal methanotrophs and related methanogens that could validate and complement findings in the literature, we performed domain based (meta)genome comparison between selected metagenomes of ANME and genomes of methanogens, as done previously for bacterial genomes [39]. For archaeal methanotrophs, we used the metagenomes of ANME-1 [40, 41], ANME-2a [42], and ANME-2d [28, 43]. For methanogens, we used genomes of closely and distantly related species able to perform acetoclastic methanogenesis (A:* Methanosaeta concilii* GP6), methylotrophic methanogenesis (M-1:* Methanococcoides burtonii* DSM6242, M-2:* Methanolobus tindarius* DSM2278, and M-3:* Methanohalophilus mahii *DSM5219), hydrogenotrophic methanogenesis (H-1:* Methanospirillum hungatei* JF-1, H-2:* Methanobacterium formicicum* DSM3637, H-3:* Methanococcus maripaludis* C5, and H-4:* Methanoregula formicica* SMSP), and both acetoclastic and methylotrophic methanogenesis (AM:* Methanosarcina acetivorans *C2A). The genome of a sulfate-reducing archaeon that contained most enzymes for methanogenesis except for Mcr (S:* Archaeoglobus fulgidus* DSM 4304) was also included in the comparison. For each dataset the protein domains were obtained through InterProScan 5.17-56.0 using the TIGRFAM, ProDom, SMART, PROSITE, PfamA, PRINTS, SUPERFAMILY, COILS, and Gene3D domain databases. Results of the analysis are given in Table 2 and Table S1 of the Supplementary Material available online at https://doi.org/10.1155/2017/1654237. Since the ANME-1 metagenome assembled by Stokke et al. 2012 [40] contained many bacterial genes, we did not refer to this data for the domain based (meta)genome comparison but only used the ANME-1 metagenome described by Meyerdierks et al., 2010 [41]. We included both ANME-1 metagenomes to analyze the organization of genes for the formaldehyde-activating enzyme (Table S2) and the iron-only hydrogenase (Table S3).

## 2. Reversal of the Methanogenesis Pathway

### 2.1. The Central Methanogenesis Pathway

**Figure 2:**
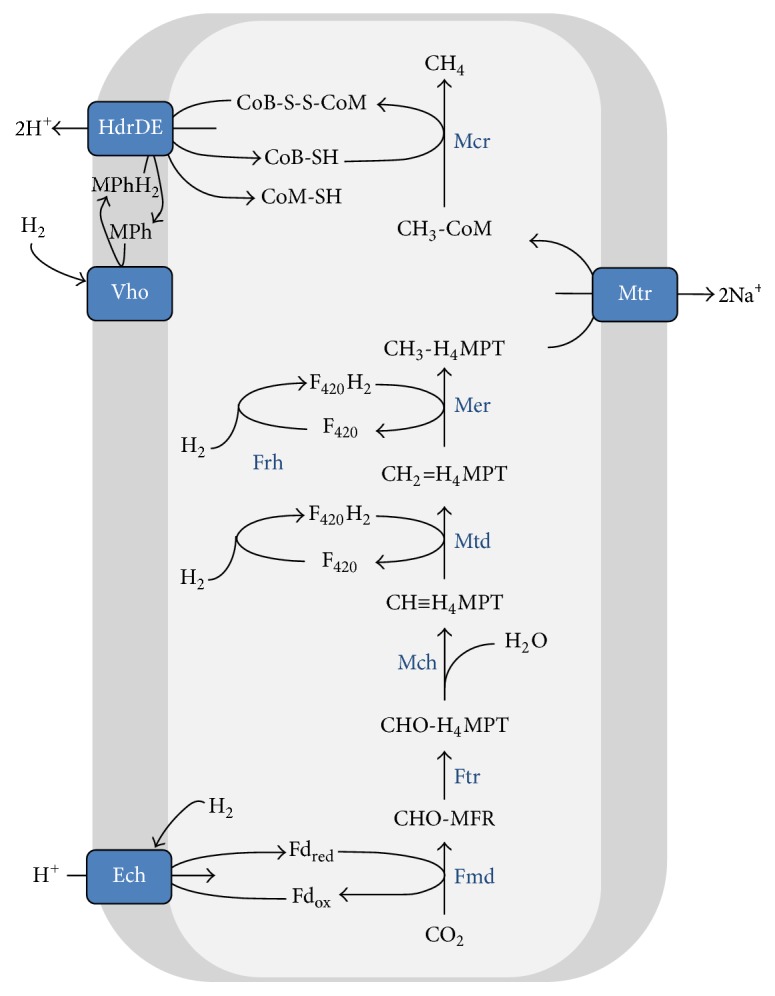
Hydrogenotrophic methanogenesis in cytochrome containing* Methanosarcina barkeri*. Black lines represent presence of conversions. See Table 3 for nomenclature.

**Figure 3:**
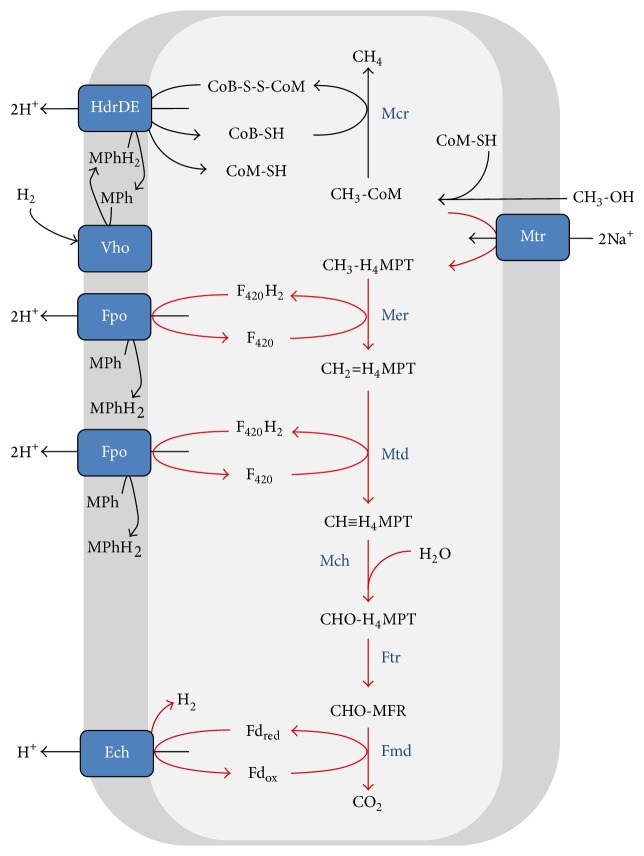
Methylotrophic methanogenesis in cytochrome containing* Methanosarcina barkeri*. Black lines represent presence of conversions and red lines indicate reversal of the hydrogenotrophic methanogenic pathway. See Table 3 for nomenclature.

ANME are described to perform “reverse methanogenesis” [44] which implies the complete reversal of methanogenesis from H_2_ and CO_2_, that is, hydrogenotrophic methanogenesis (for kinetic and thermodynamic considerations, the reader is referred to [45]). During “forward” hydrogenotrophic methanogenesis, CO_2_ is reduced to CH_4_ with reducing equivalents derived from H_2_ (Figure 2). During methylotrophic methanogenesis, this pathway is already partly reversed. Methylotrophic methanogens utilize one-carbon compounds such as methylamines, methanol or methylated sulfur compounds (methanethiol, dimethyl sulfide) that are activated to methyl coenzyme M. About 75% of the methyl coenzyme M (CH_3_-CoM) is reduced to produce CH_4_ and about 25% of CH_3_-CoM is oxidized to CO_2_ using the methanogenesis pathway in reverse during methylotrophic growth. The oxidative part provides reducing equivalents that are needed for the generation of the proton motive force in the methanogenic respiratory chain and the reduction of CH_3_-CoM by methyl coenzyme M reductase (Mcr) [46] (Figure 3). In all methanogens, the Mcr reaction operates in the forward reaction and yields methane and the heterodisulfide of coenzyme B and coenzyme M (CoB-S-S-CoM):

(1) CH3-CoM+CoB-SH⟶CH4+CoB-S-S-CoM;ΔG0=−30 kJ mol−152

**Figure 4:**
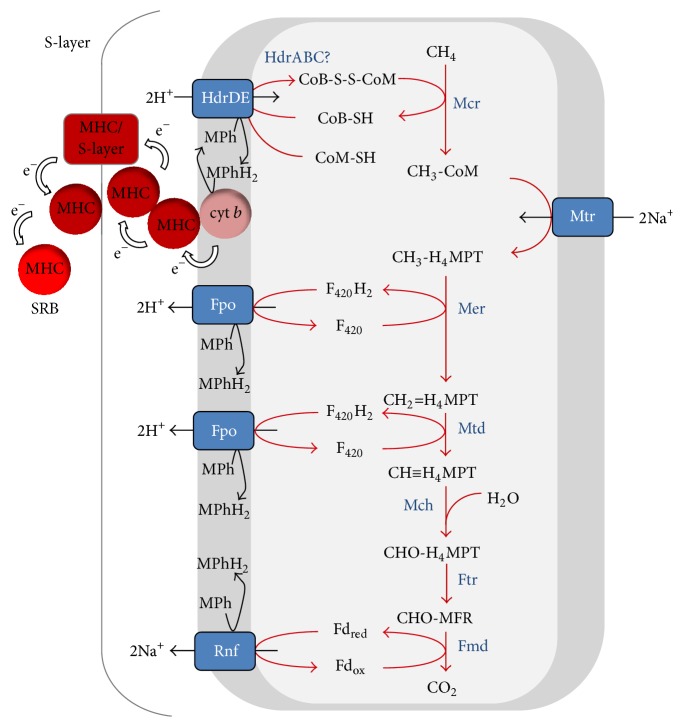
Methanotrophic pathway during S-AOM by ANME-2a [42]. Red lines indicate reversal of the hydrogenotrophic methanogenic pathway. See Table 3 for nomenclature.

**Figure 5 fig5:**
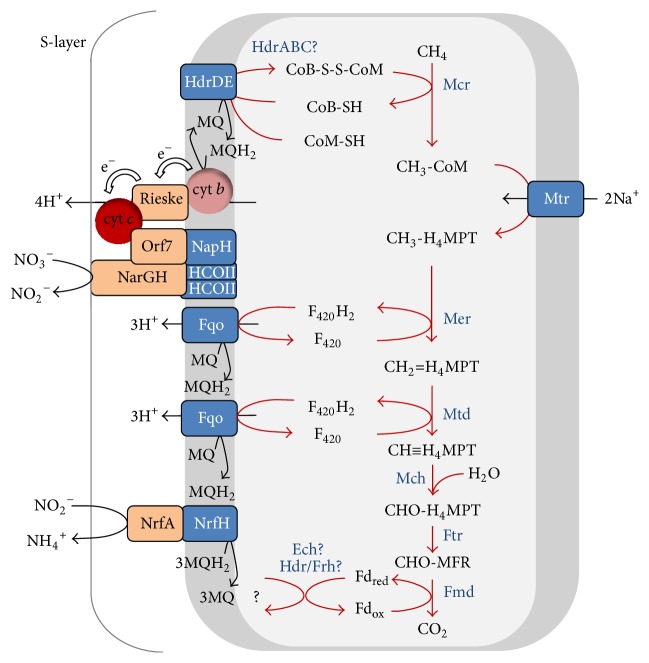
Methanotrophic pathway during N-AOM by “*Ca. *M. nitroreducens” MPEBLZ (ANME-2d) [43]. Red lines indicate reversal of the hydrogenotrophic methanogenic pathway. See Table 3 for nomenclature.

**Figure 6:**
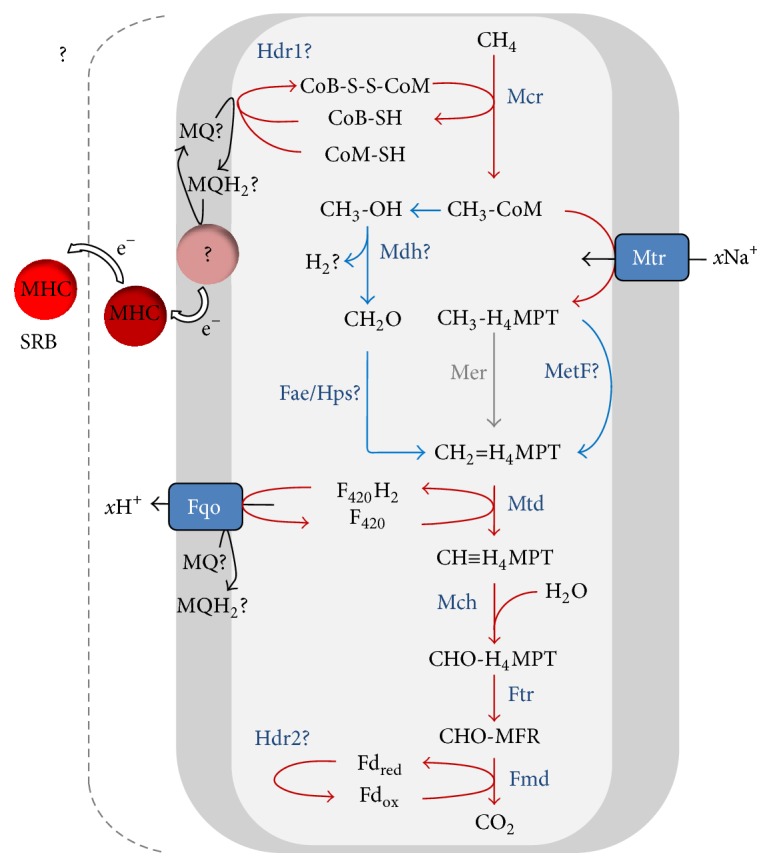
Methanotrophic pathway during S-AOM by ANME-1 [40, 41]. Red lines indicate reversal of the hydrogenotrophic methanogenic pathway, grey lines represent absence of conversions, and blue lines indicate a bypass of the hydrogenotrophic methanogenic pathway. See Table 3 for nomenclature.

The heterodisulfide is a central intermediate and acts as terminal electron acceptor in all methanogens. In hydrogenotrophic methanogens without cytochromes, it is the electron acceptor of the cytoplasmic electron-bifurcating CoB-S-S-CoM reductase (HdrABC) and F_420_-nonreducing hydrogenase (MvhADG) complex [47, 48] that is needed to provide reduced ferredoxin for the first step in methanogenesis; the reduction of CO_2_ to a formyl group. Within the methanogens with cytochromes, only a few members of the genus* Methanosarcina *are able to grow on H_2_/CO_2_. They use a ferredoxin-dependent hydrogenase (Ech) for ferredoxin reduction and an additional membrane bound methanophenazine-dependent hydrogenase (Vho) for H_2_ oxidation coupled to the reduction of the heterodisulfide by the membrane bound CoB-S-S-CoM reductase (HdrDE). F_420_ cycling can be accomplished using the F_420_-dependent hydrogenase (Frh) and F_420_H_2 _: phenazine oxidoreductase (Fpo) complex [48] (Figure 2). For methanogens it is of crucial importance that Mcr operates in the forward reaction to yield methane and the heterodisulfide. If all reactions of the methanogenic pathway are reversed such as during AOM, the pathway requires the input of energy and produces electron donors (Figures 4 [Fig fig5]–6). Therefore, during AOM, an external electron acceptor is needed which makes the reaction thermodynamically favourable (Table 1). The reverse reaction of Mcr is therefore an essential step in AOM and is discussed in Section 2.2. The respiratory chain that is needed for using different terminal electron acceptors will be discussed in Section 3.

Evidence that ANME use the reverse methanogenesis pathway during AOM is derived from metagenomic and metatranscriptomic analyses. This showed that all genes for the (reverse) methanogenic pathway were present and expressed in ANME-2a [42] (Figure 4) and ANME-2d [28] (Figure 5). ANME-1 were consistently lacking the gene encoding N^5^,N^10^-methylene- tetrahydromethanopterin (H_4_MPT) reductase (Mer), which is an enzyme needed to oxidize methyl-H_4_MPT during methane oxidation [40, 41, 44, 51] (Figure 6, Table 2). Possible explanations could be that (1) the* mer* gene is present but not yet detected, (2) ANME-1 used a bypass of this step, and (3) Mer was replaced by a structural analogue. The first possibility is highly unlikely. Although no closed genome of ANME-1 is publicly available to date, all ANME-1 metagenomes consistently only lack Mer and no other methanogenic genes. The second possibility was proposed previously where ANME-1 uses a bypass of Mer via the formation of methanol or methylamine [41], as was detected in deletion mutants of* Methanosarcina* [52, 53]. Here, CH_3_-CoM was presumably converted to methanol by a methyltransferase and subsequently to formaldehyde by a methanol dehydrogenase (Mdh). Then, formaldehyde would be converted to N^5^,N^10^ methylene-H_4_MPT using a fusion protein of the formaldehyde-activating enzyme (Fae) and a hexulose-6-phosphate synthase (Hps) [53] (Figure 6). Both Fae and Hps were found in the ANME-1 metagenome [41] and metaproteome [40, 51]. However, no genes coding for enzymes involved in methanol metabolism were detected in these ANME-1 datasets [40, 41, 51] (Table 2), indicating that this alternative pathway probably does not occur. The presence of the Fae/Hps fusion protein in ANME-1 during AOM could also be explained by its involvement in ribose phosphate synthesis and not in AOM [54]. Indeed, the Fae gene domains of ANME-1 were located in between ribulose-phosphate binding barrel and ribosomal protein S5 domains (Table S2). The third possibility of a structural analogue is most likely since an analogue of N^5^,N^10^-methylene tetrahydrofolate (H_4_HF) reductase (MetF) was expressed by ANME-1 during AOM which could replace Mer [40] (Figure 6).

### 2.2. Methyl Coenzyme M Reductase (Mcr)

The enzymatic reaction of a purified Mcr from ANME has not been measured to date. The key question is whether a methanogenic Mcr can explain the observed in situ AOM rates or if the Mcr of ANME is structurally altered. There are three main factors to be considered: the kinetic limitations as defined by enzyme properties (i.e., half-maximal activity at a specific *K*_*M*_ value), the thermodynamic constraints of the enzymatic reaction, and the maximal or ambient rate of the enzymatic reaction. For the Mcr from* Methanothermobacter marburgensis*, kinetic parameters have been determined to illustrate the reversibility of reaction (1). In the methanogenic reaction, the purified Mcr isoform I [55] catalyzes the production of methane with a *V*_max_ of 30 U mg^−1^ and a *K*_*M*_ of 5 mM for CH_3_-CoM. The same (methanogenic) enzyme was able to oxidize methane to CH_3_-CoM with a rate of 0.0114 U mg^−1^ and a *K*_*M*_ for methane of ~10 mM [56].

To answer if the observed AOM rates are in accordance with the measured methane oxidation rate for the purified Mcr enzyme from* M. marburgensis*, the Mcr activity during AOM is needed. Estimates for AOM rates in terms of activity (per cell dry mass) range between <1 and 20 mmol day^−1^ and g cell dry mass^−1^ [56–66]. This equals an activity of 0.7 to ~14 nmol min and mg cell dry mass^−1^. About half of the cell dry mass is protein, so the activity for the ANME archaea would approximate 1.4 to 28 nmol min and mg protein^−1^. To estimate the activity per mg of Mcr, the proportion of Mcr to cellular protein is needed. It was reported that 7% of the protein of ANME microbial mats from the Black Sea [67, 68] and 10.4% of peptides from Hydrate Ridge mesocosms is Mcr [51]. As these were no pure cultures, the actual percentages of Mcr in ANME cells may be higher. Transcriptome data for ANME-2d [43] showed that about 19% of the total transcripts were derived from the* mcr* genes indicating (though not demonstrating) a high Mcr content in ANME-2d. Estimating that 10% of the cellular protein would be Mcr, the specific activity of the enzyme would be between 14 and 280 nmol min and mg Mcr protein^−1^, which is up to 25 times higher than the measured reverse reaction rate of the* M. marburgensis* enzyme (~12 nmol min and mg Mcr^−1^ [56]). However, the reverse reaction rate of the* M. marburgensis* Mcr was determined under nonsaturating substrate conditions and was therefore not possibly representing the true maximum rate. Nevertheless, both reverse reaction rates are in the same order of magnitude, other than the forward reaction of 30,000–100,000 nmol min and mg Mcr^−1^. Thus, it seems that the Mcr in ANME may have similar catalytic properties as the methanogenic enzyme and that the high amount of Mcr per mg total cell biomass in ANME may in part compensates for the apparently relatively slow catalysis.

Considering the thermodynamic constraints, the Gibbs free energy change of the Mcr forward reaction under standard conditions is around −30 kJ mol^−1^ [56]. Therefore, the reverse reaction is endergonic under standard conditions and will not proceed. However, high methane concentrations (10^5^ according to reaction (1) [69, 70]) may lead to a favourable change in the Gibbs free energy in the direction of AOM. High methane partial pressure prevails at many habitats where AOM has been detected. The solubility of methane at atmospheric pressure is only 1.3 mM [71]. Consequently, increased AOM rates were reported upon pressurizing samples of different geographical origin [59, 60, 72, 73]. The *K*_*M*_ of Mcr of* M. marburgensis* for methane was determined at or above 10 mM and reported *K*_*M*_ values of S-AOM varied from (at least) 1.1 mM [74], a few mM [57], to even 37 mM (equivalent to 3 MPa CH_4_) [58]. Thus, high pressure and therefore high concentrations of methane in the natural habitat accelerate the oxidation rate of methane by Mcr. Future research to accurately determine *K*_*M*_ values and rates for Mcr at different methane partial pressures is however needed. This may seem difficult, but microbial activity measurements at in situ methane partial pressure were shown to be successful in the laboratory [75].

It was suggested that the Mcr reaction is the rate limiting step in reverse methanogenesis [56] which is in line with the above described challenges. Supporting these findings, there does not seem to be a major change in the amino acid structure that determines whether the backwards or the forward reaction of Mcr is favoured. Amino acid alignments [67] and the crystal structure of ANME-1 Mcr [76] indicated high overall similarity of the methanogenic and methanotrophic enzyme and unambiguously demonstrated that CoM-SH and CoB-SH are substrates of the methanotrophic enzyme. However, several posttranslational modifications of amino acids were different between methanogens and ANME archaea, and the cofactor F_430_ (the prosthetic group of Mcr) is modified in ANME-1, but not in ANME-2 or ANME-3 archaea [51, 63, 67, 68, 76, 77]. Furthermore, ANME-1 seems to lack the noncatalytic protein D domain of the* mcr *gene that is present in all other methanogens and methanotrophs but of which the function is unknown (IPR003901, Table 2) [51]. A metabolically engineered* Methanosarcina acetivorans *was able to convert methane and CO_2_ to acetate with a plasmid containing Mcr derived from ANME-1 [78]. It is thus unclear if only thermodynamic constraints and the abundance of Mcr ensure AOM activity, or if also specific modifications can have an effect on the reverse activity of Mcr.

### 2.3. Methane Oxidation by Methanogens

Pure cultures of methanogens were not able to oxidize methane under high methane and low hydrogen concentrations (reviewed in [79, 80]). Methanogens are only able to oxidize methane during net methane production [81]. Labeled methane addition (^13^C or ^14^C) to pure cultures of methanogens showed production of labeled CO_2_ during net methane production. This characteristic was confirmed with several pure cultures of methanogens [82–84]. The process was called “trace methane oxidation” (TMO), since the CO_2_ was formed in trace amounts relative to the methane produced [83]. It is not clear if TMO is due to the reported reversibility of individual enzymes [66], or if it is an active microbial process from which energy can be conserved. TMO was speculated to be an active metabolic process for three reasons: (1) the amount of methane oxidized varied between different species of methanogens grown on the same methanogenic substrate; (2) the amount of methane oxidized varied between different methanogenic substrates; and (3) TMO products varied between different methanogenic substrates [83, 84]. For instance, when grown on acetate,* Methanosarcina acetivorans* produced labeled acetate from labeled methane. When grown on carbon monoxide, it produced both labeled acetate and methyl sulfides from labeled methane [84]. During hydrogenotrophic and methylotrophic methanogenesis, TMO mainly produced CO_2_ from labeled methane [83]. However, in contrast with AOM, TMO rates never exceeded methanogenesis rates, even during long-term incubation with methane and sulfate [85]. It seems that methanogens are not able to conserve energy from TMO, even under thermodynamically favourable conditions. TMO occurs both in absence and presence of an external electron acceptor and only during net methanogenesis. It is therefore most likely caused by the reported back flux of individual enzymes of the methanogenic pathway [66].

TMO also occurred in granular sludge and in freshwater and terrestrial samples. These mixed communities showed higher TMO rates than pure cultures, reaching up to 90% of the methane produced [27, 85–87]. TMO should therefore be carefully considered in the experimental setup and interpretation of results when studying AOM in environmental samples, especially since TMO rates were, like AOM, stimulated by a high methane partial pressure [72, 86, 88]. Sulfate reduction was also stimulated by higher methane partial pressures [85]. Thus, a high methane partial pressure can have a stimulating effect on methane oxidation (either through AOM or TMO) and SR, which could be unrelated to S-AOM. Moreover, addition of iron sulfate (FeSO_4_) or manganese oxide (MnO_2_) also increased TMO rates [86]. Therefore, methane-dependent SR and sulfate- or metal-dependent methane oxidation are not necessarily indications for AOM in mixed cultures. In conclusion, when studying complex “black-box” communities, only net methane oxidation is proof for AOM activity.

### 2.4. Methane Production by ANME

The process of S-AOM is at the energetic limit for sustaining life, with estimates of Gibbs free energy yields between −18 and −35 kJ mol^−1^ [45, 79, 89–91] and doubling times between 1.1 and 7.5 months [65, 72, 73, 92, 93]. Since S-AOM operates close to its thermodynamic equilibrium, the reversibility of individual enzymes leads to measurable back flux, producing methane (3–7% of AOM) and sulfate (5.5–13% of SR) during S-AOM [66]. This “trace methane production” was observed in situ [11] and in sediment slurries, with methanogenesis around 10% [62, 94] or even as high as 50% of AOM [34]. When sulfate becomes depleted, Gibbs free energy yields become even lower (less negative) and the enzymatic back flux becomes even more apparent, up to 78% of net AOM [95]. Previous measurements of ^13^C depletion below the sulfate-methane transition zone (SMTZ) of marine sediments that were thought to be indicative for methanogenesis could therefore instead be attributed to the back flux of AOM [95]. The occurrence of ANME-1 without a bacterial partner in sediment layers where sulfate was depleted was previously interpreted as evidence that ANME-1 perform methanogenesis [24], but in light of the above it could also indicate AOM. There are indeed reports of AOM activity below the SMTZ in the methanogenic zone [96–99]. In contrast, AOM with other electron acceptors than sulfate operates far from the thermodynamic equilibrium with Gibbs free energy changes between −517.2 and −841.4 kJ mol methane^−1^ (Table 1). Here, the reported anaerobic back flux [66] is expected to be less apparent.

In laboratory incubations, researchers were not able to stimulate net methanogenesis through addition of methanogenic substrates to AOM performing sediments [62, 94]. In two cases, researchers were successful [23, 100]. In one of these cases, sediment-free long-term AOM enrichments that were dominated by ANME/SRB were incubated with methanogenic substrates. The resulting methanogenic activity most likely came from the enrichment of a minor population of methanogens (up to 7‰ of total archaeal gene tag sequences) that was present in the inoculum [100]. In the second study, methanogenic substrates were added to ANME-1 and ANME-2 dominated microbial mat samples and methanogenesis also occurred [23]. However, no information was provided for the total archaeal community composition, which makes it impossible to exclude methanogens as the responsible organisms.

Genomic information of ANME also gives indication on potential methanogenic routes. Considering methylotrophic methanogenesis, no gene homologues catalyzing methyl transfer from methylated substrates to coenzyme M were detected in ANME (Table 2) [40–43]. Acetoclastic methanogenesis needs either the AMP- and ADP-forming acetyl-coenzyme A synthetase (Acs and Acd, resp.) or proceeds via acetate kinase and phosphotransacetylase. In ANME-1, only the alpha subunit of a homologue of Acd was expressed during AOM [41], but in an ANME-1 proteome of active AOM biomass, no Acd was detected [40]. The Acd gene was detected in the single-aggregate genome and transcriptome of ANME-2a [42] and in ANME-2d [43]. However, gene domains for Acd are also present in methanogens unable to use acetate as substrate (Table 2) and are probably used for lipid metabolism. In hydrogenotrophic methanogenesis, hydrogenases are used to replenish reduced coenzyme B and to recycle oxidized F_420_ (discussed in Section 2.1). Both the cytoplasmic Mvh complex and the membrane bound Vho were not present in ANME-2d [43] and not expressed in ANME-2a (which also lacked Ech and F_420_-dependent hydrogenase (Frh)) [42], making hydrogenotrophic methanogenesis unlikely. In ANME-1, both the cytoplasmic HdrABC and MvhD are present, as well as homologues of Frh and Ech, but these were lacking catalytic subunits [40, 41]. An iron hydrogenase was found in both ANME-1 metagenomes but not in any other methanotroph or methanogen [41] (Table 2). This iron hydrogenase domain is part of a gene that is 70% identical to a [FeFe]-hydrogenase of* Dehalococcoides mccartyi*. [FeFe]-hydrogenases catalyze reversible H_2_ production and uptake, but these were presumed to have no key function in AOM [41]. However, the gene is part of a gene cluster of three genes containing a 51 kDa NADH: ubiquinone oxidoreductase subunit (Table S3), which could potentially form a complex that generates a proton motive force during hydrogen oxidation. Therefore, hydrogenotrophic methanogenesis by ANME-1 cannot be excluded yet.

## 3. Respiration during Anaerobic Oxidation of Methane

For net AOM to occur, an external electron acceptor is needed which results in a favourable Gibbs free energy change (Table 1). A variety of terminal electron acceptors have been discovered for AOM which will be discussed in Sections 3.1–3.3.

### 3.1. Sulfate-Dependent AOM

During sulfate-dependent AOM, electrons are transferred from ANME to the sulfate-reducing bacterial partner. Previous work tried to uncover how electrons were transferred and most compounds that could act as interspecies electron carrier (IEC) were excluded to be involved in AOM, such as methanol, hydrogen, methanethiol, acetate, and carbon monoxide [57, 61, 62, 94, 101]. Indications that polysulfide could act as IEC were found, and ANME-2a archaea were thought to perform both AOM and sulfate reduction (SR) [102]. However, in marine seeps, hydrothermal vents, and other nondiffusion based sediments, AOM rates are high and ANME form close associations with SRB in dense aggregates [1, 5, 103, 104]. In these aggregates, the high AOM rates could not be explained by diffusion of an IEC, which made direct interspecies electron transfer (DIET) a more plausible mechanism [89, 105, 106]. Cellular activities were independent of aggregate type and distance between the syntrophic partners within the aggregate, which is best explained by DIET [107]. DIET is normally achieved using multiheme cytochrome c proteins (MHCs) and conductive pili (i.e., nanowires) which are mainly found in bacteria that donate electrons extracellularly, such as* Geobacter* and* Shewanella* species [108–114]. Indeed, ANME-2a from seep-sediment samples seem to transfer electrons directly using large MHCs [107], which were found in the metagenome of ANME-2a [107, 115]. ANME-1 and the associated bacterial partner also overexpressed genes for extracellular MHCs during AOM [116], which complements previous findings of transcription [41] and translation [40] of ANME-1 related MHC genes. Domain based (meta)genome analysis shows the high abundance of MHC domains in different ANME as compared to methanogens (Table 2). A recently isolated bacterial partner of ANME-1 (“HotSeep-1”) also produced cell-to-cell connections using pili-derived nanowires [116], which explain previously detected* Deltaproteobacteria*-related pili genes in an AOM sample dominated by ANME-1 [40].

How ANME use MHCs to donate electrons to the bacterial partner is not yet clear. For ANME-2a, the electrons probably flow from methanophenazine to membrane integrated di-heme cytochromes (cytochrome *b*/*b*_6_) that transfer the electrons through the S-layer via MHC/S-layer fusion proteins to extracellular MHCs (Figure 4) (Figure 4 in [107]). Exosortases and archaea-specific archaeosortases are involved in export of cell surface proteins, such as the archaeal S-layer proteins. These transpeptidases recognize specific signal peptides for protein- sorting; that is, archaeosortase A recognizes the protein-sorting signal PGF-CTERM and archaeosortase C recognizes the PEF-CTERM signal [117]. Both ANME-2a and 2d show presence of di-heme cytochromes, archaeosortase A (IPR014522), archaeosortase C (IPR022504), and other exosortase gene domains (Table 2). Moreover, some genes of both ANME-2a and 2d contained both MHC and PGF or PEF-CTERM domains. Lastly, some genes of both ANME-2a and 2d contained both MHC and S-layer domains [107], indicating that these could form the above-stated MHC/S-layer fusion proteins.

ANME-1 do not seem to have di-heme cytochromes (Table 2) [115]. PGF related domains (IPR026453 and IPR026371) were present in all ANME but PEF-CTERM (IPR017474) related domains were absent in ANME-1 (Table 2). Moreover, ANME-1 lacked archaeosortase A (IPR014522) and archaeosortase C gene domains (IPR022504), as well as some other exosortases (Table 2). A search in NCBI's conserved domains database (CCD, [118]) and the EMBL InterPro database [119] of amino acids sequences of all genes from ANME metagenomes that contain MHC domains showed that ANME-1 did not have any protein-sorting signal or S-layer domains within these genes. In fact, S-layer domains were completely absent in ANME-1 (Table 2). These results imply that ANME-1 do not use di-heme cytochromes for electron transfer to MHCs and do not produce an S-layer (Figure 6). This implies that ANME-1 use a different mechanism for DIET and could explain the need for less MHCs by ANME-1 (Table 2) and the observed pili-derived nanowires produced by the bacterial partner [116]. The genome of the bacterial partner of ANME-1 (“HotSeep-1”) encoded 24* c*-type cytochromes of which 10 were similar to secreted MHCs of* Geobacter sulfurreducens *[120] which also uses pili for DIET [121].

In the case of ANME-2a, it is not clear if pili (i.e., nanowires) were formed during AOM. It was previously thought that electrically conductive pili seemed to be a prerequisite for current production and DIET [122, 123], even when syntrophs were closely associated [124] such as within ANME-2/SRB aggregates. However, conductive materials such as granular activated carbon were shown to be able to substitute pili in DIET [124]. Although in previous work conductive materials such as phenazines or humic acids did not seem to stimulate AOM rates [61], in a recent study AOM was decoupled from SR using artificial electron acceptors [125]. This indicates that conductive materials can indeed replace pili and that ANME-2a/b could possibly couple AOM to metal oxide reduction or any other suitable electron acceptor (discussed in Section 3.3). However, it needs to be proven if in ANME-2/SRB aggregates no pili are formed and if the mechanism of DIET is fundamentally different from ANME-1.

As for the polysulfide shuttling theory [102], canonical genes for dissimilatory sulfate reduction such as adenylyltransferase (Sat), APS reductase (Apr), and dissimilatory sulfite reductase (Dsr) which are all present in the sulfate-reducing archeon* A. fulgidus* (Table S2) were not found in metagenomes of ANME-1 [41] and ANME-2a (Table S1). The enzymes Sat and Dsr were also not detected in ANME cells using fluorescent immunolabelling [126]. ANME-1 were previously found to encode most proteins for assimilatory sulfate reduction [41]. ANME-2d only harbor gene domains that encode Sat and assimilatory ATP sulfurylase, APS kinase, and sodium/sulfate symporters, which were not present in ANME-2a (Table S1). It is therefore clear that at least ANME-2a cannot donate electrons to sulfate but need to donate electrons to a sulfate-reducing partner.

### 3.2. Nitrate-Dependent AOM

Unlike during S-AOM, ANME-2d that perform N-AOM do not need a bacterial partner but transfer electrons directly to a membrane bound nitrate reductase (Nar) [28, 43] (Figure 5). The ANME-2d genomes contain most MHCs found so far in archaea [107, 115] (Table 2). Of the 87 proteins that contained a CxxCH binding motif, of which 43 seemed to be true* c*-type cytochromes [115], 23 CxxCH motif-encoding transcripts were expressed during N-AOM [43]. The function of most of these MHCs is not known, but they are likely involved in nitrate reduction since* c*-type cytochromes are capable of operating in the wide range of redox potentials that couple nitrate reduction (*E* ^0′^ (NO_3_^−^/NO_2_^−^) = +433 mV) and methane oxidation (*E* ^0′^ (CoM-S-S-CoB/CoM-SH+CoB-SH) = −143 mV) [43]. Nitrate as terminal electron acceptor in anaerobic respiration has been found in halophilic and thermophilic archaea [127]. The* nar* gene cluster of “*Ca.* M. nitroreducens” comprises several genes including the catalytic alpha (NarG, molybdopterin) and beta (NarH, iron sulfur cluster) subunit of nitrate reductase [28, 43]. The (halo)archaeal nitrate reductase complex was reported to be located at the extracellular side of the cytoplasmic membrane [128] and in most archaea associated with the cytoplasmic membrane via NarM [129]. The “*Ca. *M. nitroreducens” genome does not encode NarM but encodes a TAT signal peptide at the N-terminus of NarG for translocation across the cytoplasmic membrane [28, 43]. Interestingly, the NarG and NarH seem to have been acquired from the Proteobacteria via lateral gene transfer [28].

It is not yet clear at which point in the metabolism “*Ca.* M. nitroreducens” conserves energy. During reverse methanogenesis, N^5^-methyl-H_4_MPT:CoM methyltransferase (Mtr) dissipates the sodium ion potential across the cytoplasmic membrane so subsequent steps in N-AOM have to be coupled to the build-up of a proton or sodium motive force to make the overall process energetically favourable. The analysis of an environmental genome [43] indicated presence of several protein complexes involved in electron transport and energy conservation. Electrons that enter the respiratory chain could be transported by membrane-integral electron carriers (i.e., menaquinones) to a Rieske-cytochrome* b* complex that may use cytochrome* c* as electron acceptor. This in turn may be the electron donor for the unusual nitrate reductase complex. Energy conservation is thermodynamically and mechanistically feasible at the F_420_H_2_ dehydrogenase and the Rieske-cytochrome* b *complex (Figure 5) (Figure 2 in [43]). Further investigations are needed to determine whether nitrate reductase is also involved in energy conservation, but this working hypothesis is strengthened by the presence of cupredoxin, multicopper oxidase domains, and copper centers related to the periplasmic domain of cytochrome* c* oxidase subunit II (HCO II) in ANME-2d (Table S1).

Both ANME-2d genomes discussed here are derived from bioreactors where “*Ca.* M. nitroreducens” formed syntrophic cultures with nitrite scavenging bacteria, either with* “Ca. *Kuenenia stuttgartiensis” (anammox bacteria) [28] or* “Ca. *Methylomirabilis oxyfera” (NC10 bacteria) [31, 43]. This indicated that ANME-2d could be dependent on those bacteria for nitrite removal. However, in addition to nitrite, “*Ca.* M. nitroreducens” may also produce ammonium during AOM by a pentaheme* c*-type nitrite reductase (NrfAH) encoded in the genome [43] (Figure 5). In fact, both ANME-2d genomes contain domains for NrfA (IPR003321) and NrfH (IPR017571) (Table S1) implying that both ANME-2d species are not necessarily dependent on a nitrite scavenger during AOM.

### 3.3. Metal-Dependent AOM

Evidence for metal-dependent AOM was found in marine sediments [130–132]. In nonmarine environments, AOM was also hypothesized to be coupled to iron and/or manganese oxide reduction [26, 133–136] or even coupled to the reduction of humic acids [136, 137]. However, organisms responsible for metal-dependent AOM were not identified in these studies. It was speculated that JS1 bacteria, methanogenic archaea, and* Methanohalobium*/ANME-3 could be responsible for iron-dependent AOM [138]. Other researchers speculated that either ANME-1 or* Methanococcoides*/ANME-3 together with a bacterial partner were responsible for manganese-dependent AOM [139]. In another study where AOM was decoupled from SR, ANME were not detected, which leaves open the possibility that other archaeal clades besides ANME could perform metal-dependent AOM [131].

It was recently observed that cultures containing ANME-2a and ANME-2c could decouple AOM from SR in the presence of artificial electron acceptors, humic acids, and soluble iron [125], which confirmed previous findings of AOM not connected to SR in ANME dominated samples [140]. This suggests that ANME-2 could also use insoluble metal oxide minerals as electron acceptor during AOM. The MHCs of ANME-2a/b and ANME-2d are larger than those in* Shewanella* and* Geobacter* species [107], which are known to be capable of extracellular electron conduction. It was speculated that both ANME-2d and* Ferroglobus placidus*, of which the latter can perform solid iron reduction, can fold CxxCH motifs into extracellular conductive structures or pili [115]. Many of the MHCs of ANME-2d were not expressed when grown with nitrate (discussed in section 3.2), implying that these are not needed for nitrate reduction [43]. This strengthens the hypothesis that also ANME-2d could couple AOM to reduction of other extracellular electron acceptors than nitrate and even to insoluble metal oxides. Indeed, recent work showed that ANME-2d could be involved in AOM coupled to chromium(VI) reduction [141] (Table 1, reaction (5)) and in AOM coupled to the reduction of soluble iron and insoluble ferrihydrite and birnessite minerals [142] (Table 1, reactions (3) and (4)). ANME-2d could even possibly donate electrons to a bacterial partner: besides nitrate-rich environments, ANME-2d archaea have been found in wells of an aquifer where sulfate and methane concentrations overlap [143]. Moreover, ANME-2d was the only clade detected in sediments of a freshwater lake where S-AOM occurred [144]. Sulfate concentrations in these studies were low, but above 1 mM and thus higher than the lowest reported concentrations for S-AOM to occur [145, 146]. Lastly, sequences of ANME-2d were relatively more abundant in freshwater sediments fed with methane and sulfate than in sediments fed with only methane or only sulfate and no N-AOM activity was detected when fed with nitrate and methane [27]. These indications hold promise that direct experimental evidence for sulfate-dependent AOM by ANME-2d could be found in the future.

The ANME-1 genome contains fewer MHC gene domains as compared to ANME-2a and ANME-2d and some other archaea, such as some methylotrophic methanogens (Table 2) and some members of the Archaeoglobales [115]. The MHCs of ANME-1 also have a smaller heme count as compared to other ANME and some other archaea, with the largest being an octaheme cytochrome [107]. Each heme within a MHC has its own redox potential and therefore structurally different MHCs represent a large range of redox potentials that can be used for bioenergetic electron transfer (reviewed in [147]). For instance, metal oxide reduction in* Shewanella oneidensis* MR-1 is catalyzed by a chain of a tetraheme (CymA), two decaheme (MtrA and MtrB), and eventually extracellular decaheme cytochromes OmcA/MtrC that reduce the iron minerals [121, 148, 149]. In* Geobacter sulfurreducens*, iron mineral reduction seems to be catalyzed by the tetraheme cytochrome OmcE and hexaheme cytochrome OmcS transferring electrons from the outer membrane to type IV pili that transmit the electrons to iron minerals [121, 150, 151]. Since ANME-1 lack MHCs of the correct size and lack gene domains for pili production [116], they seem unable to reduce minerals via both mechanisms present in* Shewanella *and* Geobacter*. It can therefore be speculated that ANME-1 are less versatile in electron acceptor use and are not able to reduce solid metal oxides. However, DIET mechanisms are still not well understood and the true differences between MHCs of different ANME types need to be investigated using biochemical methods. This would allow uncovering the true capabilities concerning DIET.

### 3.4. Menaquinones and Methanophenazines

ANME-2a encoded a protein with a domain specific for PhzF, which is an enzyme involved in phenazine biosynthesis in* Pseudomonas fluorescens* [152, 153]. The respective gene is not present in all methanophenazine containing methanogens (in our genome comparison only in* M. acetivorans* and* M. formicica*, Table 2) so it is unclear whether this enzyme is really involved in methanophenazine biosynthesis. It is however likely that ANME-2a use methanophenazines in their respiratory chain since gene domains for menaquinone biosynthesis were absent (Table 2). “*Ca*. M. nitroreducens” probably uses menaquinones as membrane-integral electron carrier since environmental genomes [28, 43] encoded the futalosine (mqn) biosynthesis pathway as reported for* Archaeoglobus fulgidus *[154] (Table 2). Moreover, “*Ca*. M. nitroreducens” enrichment cultures showed absence of methanophenazines [43]. ANME-1 also contained gene domains for menaquinone biosynthesis via the futalosine (mqn) biosynthesis pathway (Table 2). Indications for a quinone biosynthesis pathway in ANME-1 were previously found to be weak since only some of the Ubi homologues of the oxic ubiquinone biosynthesis pathway were detected [41], but the futalosine (mqn) biosynthesis pathway was overlooked in that particular analysis. Additionally, ANME-1 have Fqo homologues similar to* Archaeoglobus fulgidus *[44] and expressed the catalytic subunit FqoF [41]. However, since the phenazine biosynthesis domain PhzF was also present in the ANME-1 genomes (Table 2) and Fpo and Fqo are homologues, we cannot conclude on genomic information alone which redox shuttle is used by ANME-1 during AOM. If ANME-1 would use menaquinones, this would have implications for subsequent electron transfer to MHCs since methanophenazine (*E* ^0^ = −165 mV) [155] and menaquinone (*E* ^0^ = −80 mV) [156] have different redox potentials.

### 3.5. Cell Adhesion

Some gene domains involved in cellular adhesion were more abundant in ANME than in methanogens (Table 2), especially in ANME-2a and ANME-1 that are known to form syntrophic interactions for electron transfer during respiration. These domains include HYR (IPR003410) and CARDB domains (IPR011635) that both have a direct role in cellular adhesion [157] (Table 2). Interestingly, also domains related to the cellulosome of* Clostridium* species, termed dockerin and cohesin, were highly abundant in both ANME-1 and ANME-2a as compared to ANME-2d and methanogens, together with many carbohydrate binding domains (Table 2). In* Clostridium* species, dockerin and cohesin form an anchor to the bacterial cell wall that contains a scaffold with cellulose-degrading enzymes and a carbohydrate binding module that binds cellulose, altogether forming the cellulosome [158]. These domains have been found in all domains of life irrespective of cellulose utilization, but the function of such proteins outside of the cellulosome is not known [159]. Therefore, dockerin, cohesin, and carbohydrate binding domains in ANME could hypothetically form a construct that binds to carbohydrates and could possibly have a function in cell-to-cell contact or MHC adhesion, but this needs further investigation.

## 4. Future Challenges

Advances in (meta)genomics, transcriptomics, and proteomics have produced the valuable metabolic blueprints of different ANME with hypotheses on how central metabolism, electron transport, and energy conservation may function. Future experiments are needed to biochemically demonstrate that these hypotheses are correct.

The bottleneck for biochemical studies of ANME is the lack of pure cultures due to the slow and syntrophic growth. However, the recent milestone discovery of direct electron transfer provides opportunities to grow ANME, as stated previously, on electron accepting electrodes (i.e., anodes) [160]. In this way, pure or highly enriched cultures of different ANME without their respective syntrophic partner could be obtained and MHCs responsible for electric conduction could be biochemically characterized. It seems that ANME-1 are limited by the size and abundance of MHCs which could relate to differences in behavior on an anode. The partner bacteria of ANME-1 and ANME-2 could be investigated on an electron donating electrode (i.e., cathode), with specific focus on the ability to produce pili (i.e., nanowires). Also worth investigating is if besides ANME-2d, ANME-2a/b could also use insoluble electron acceptors and if ANME-2d could donate electrons to a bacterial partner.

Another future challenge is to isolate and characterize Mcr from different ANME clades. It needs to be investigated if Mcr abundance in ANME cells compensates for the slow reverse activity or if modifications in the Mcr of ANME-1 also contribute to a better reverse activity of Mcr. Moreover, the effect of methane partial pressure on reaction rates and enzyme kinetics needs to be determined in situ.

ANME-1 are potentially able to perform hydrogenotrophic methanogenesis due to the presence of a hydrogenase in the genome. Genetic indications of menaquinones as electron carrier in ANME-1 and the various cellulosome and cell adhesion related gene domains in all ANME are also topics that could be explored further. Methanogenic archaea are likely not able to perform AOM, but additional studies on TMO and more genetic modifications to stimulate AOM in methanogens could help in understanding the parameters needed for AOM to occur. Ultimately, physiological understanding of ANME will help to explain the observed ecological niche separation of different ANME clades and the occurrence of ANME without a bacterial partner. This would greatly enhance our knowledge of the methane cycle in anoxic environments.

## Supplementary Material

This supplementary material contains data on domain based (meta)genome comparison of selected metagenomes of methanotrophs and genomes of selected archaea that belongs to the article “Reverse methanogenesis, and respiration in methanotrophic archaea” by Peer H.A. Timmers, Cornelia U. Welte, jasper J. Koehorst, Caroline M. Plugge, Mike S. M. Jetten and Alfons J.M. Stams.

## Figures and Tables

**Table 1:** Gibbs free energy changes under standard conditions (Δ*G* ^0^) for anaerobic methane oxidation coupled to different electron acceptors (possibly) performed by ANME.

Reaction	Gibbs free energy (Δ*G* ^0^, kJ mol^−1^)
(1) CH_4_ + SO_4_^2−^ → HCO_3_^−^ + HS^−^+ H_2_O	−16.3
(2) CH_4_ + 4 NO_3_^−^ → HCO_3_^−^ + 4 NO_2_^−^ + H_2_O + H^+^	−517.2
(3) CH_4_ + 8 Fe(OH)_3_ + 16 H^+^ → CO_2_ + 8 Fe^2+^ + 22 H_2_O	−571.2
(4) CH_4_ + 4 MnO_2_ + 8 H^+^ → CO_2_ + 4 Mn^2+^ + 6 H_2_O	−763.2
(5) CH_4_ + 4/3 Cr_2_O_7_^2−^ + 32/3 H^+^ → 8/3 Cr^3+^ + CO_2_ + 22/3 H_2_O	−841.4

**Table 2:** Domain based (meta)genome comparison of selected metagenomes of methanotrophs and selected genomes of other archaea. Domain abundance in every (meta)genome is indicated by numbers. S-AOM performing ANME include ANME-1-s [40], ANME-1-m [41], and ANME-2a [42]. N-AOM performing ANME include ANME-2d-h [28] and ANME-2d-a [43]. The acetoclastic (A) and methylotrophic (M) methanogens include *Methanosarcina acetivorans *C2A (AM), *Methanosaeta concilii* GP6 (A), *Methanococcoides burtonii* DSM6242 (M-1), *Methanolobus tindarius* DSM2278 (M-2), and *Methanohalophilus mahii *DSM5219 (M-3). Hydrogenotrophic methanogens (H) include *Methanospirillum hungatei* JF-1 (H-1), *Methanobacterium formicicum* DSM3637 (H-2), *Methanococcus maripaludis* C5 (H-3), and *Methanoregula formicica* SMSP (H-4). The sulfate-reducing archaeon (S) is *Archaeoglobus fulgidus* DSM 4304.

	InterPro ID	ANME-1 S	ANME-1 M	ANME-2a	ANME-2d H	ANME-2d A	AM	A	M-1	M-2	M-3	H-1	H-2	H-3	H-4	S
*Central pathway*																
Mcr, alpha subunit, N-terminal	IPR003183	4	2	1	1	1	1	1	1	1	1	1	2	1	2	0
Mcr, alpha subunit, N-terminal subdomain 1	IPR015811	2	3	1	1	1	1	1	1	1	1	1	2	1	2	0
Mcr, alpha subunit, N-terminal subdomain 2	IPR015823	2	2	2	2	2	2	2	2	2	2	2	4	2	4	0
Mcr, alpha subunit, C-terminal	IPR009047	3	2	1	1	1	1	1	1	1	1	1	2	1	2	0
Mcr, alpha/beta subunit, C-terminal	IPR008924	6	4	2	2	2	2	2	2	2	2	2	4	2	4	0
Mcr, alpha/beta subunit, domain 2, C-terminal	IPR022681	9	6	3	3	3	3	3	3	3	3	3	6	3	6	0
Mcr, beta subunit	IPR003179	1	2	1	1	1	1	1	1	1	1	1	2	1	2	0
Mcr, beta subunit, C-terminal	IPR022679	3	2	1	1	1	1	1	1	1	1	1	2	1	2	0
Mcr, beta subunit, N-terminal	IPR022680	4	2	1	1	1	1	1	1	1	1	1	2	1	2	0
Mcr, gamma subunit	IPR003178	14	8	4	4	4	4	4	4	4	4	4	8	4	8	0
Mcr, protein C	IPR007687	2	1	1	1	1	1	1	1	1	1	1	1	1	1	0
Mcr, protein C-like	IPR026327	5	2	2	2	2	2	2	1	2	2	2	2	2	1	0
Mcr, protein D	IPR003901	0	0	3	6	6	3	3	3	3	3	3	9	3	6	0
Mcr, ferredoxin-like fold	IPR009024	12	6	3	3	3	3	3	3	3	3	3	6	3	6	0
5,10-methylenetetrahydromethanopterin reductase	IPR019946	0	0	2	1	1	1	1	1	1	1	1	1	1	1	1
*Acetoclastic methanogenesis*																
CO dehydrogenase/acetyl-CoA synthase complex alpha subunit	IPR004460	0	1	1	1	1	3	1	1	1	1	1	1	1	1	2
CO dehydrogenase/acetyl-CoA synthase complex beta subunit	IPR004461	13	4	2	2	2	4	4	2	2	2	2	2	2	4	2
CO dehydrogenase/acetyl-CoA synthase delta subunit	IPR004486	5	1	1	1	1	2	1	1	1	1	1	1	1	1	1
CO dehydrogenase/acetyl-CoA synthase delta subunit, TIM barrel	IPR016041	11	2	5	3	2	4	2	2	3	3	3	2	2	5	2
CO dehydrogenase b subunit/acetyl-CoA synthase epsilon subunit	IPR003704	3	2	2	2	2	4	2	2	2	2	2	2	2	2	4
*Methylotrophic methanogenesis*																
Methyltransferase MtaA/CmuA	IPR006360	0	0	0	0	0	7	0	4	5	6	0	0	1	0	0
Methanol-cobalamin methyltransferase, B subunit	IPR021079	0	0	0	0	0	3	0	2	2	2	0	0	0	0	0
Monomethylamine methyltransferase MtmB	IPR008031	3	0	0	0	0	12	0	12	21	15	0	0	0	0	0
Trimethylamine methyltransferase	IPR010426	11	0	0	0	0	4	0	5	2	2	0	0	0	0	0
Dimethylamine methyltransferase MtbB	IPR012653	0	0	0	0	0	9	0	6	6	6	0	0	0	0	0
Trimethylamine methyltransferase, *Methanosarcina*	IPR012740	0	0	0	0	0	2	0	1	1	1	0	0	0	0	0
Methylamine methyltransferase corrinoid protein reductive activase	IPR026339	0	0	0	0	0	4	0	2	2	2	0	0	0	0	0
*C-type cytochromes*																
Di-haem cytochrome, transmembrane, nitrate reduction	IPR016174	2	0	4	3	7	4	0	0	4	0	1	1	0	0	1
Doubled CXXCH motif	IPR010177	33	0	3	2	3	0	0	0	0	0	0	0	0	0	0
Cytochrome c-like domain	IPR009056	2	0	6	0	17	15	0	4	8	6	0	0	0	0	4
Class III cytochrome C (tetraheme cytochrome)	IPR020942	2	0	0	3	3	0	0	0	0	0	0	0	0	0	0
Tetraheme cytochrome domain, flavocytochrome c3 (*Shewanella*)	IPR012286	4	0	4	5	4	2	0	0	1	0	1	0	0	0	2
Octaheme c-type cytochrome	IPR024673	1	0	2	0	0	0	0	0	2	2	0	0	0	0	1
Methanogenesis multiheme c-type cytochrome	IPR027594	0	0	1	0	0	1	0	1	1	1	0	0	0	0	0
Multiheme cytochrome	IPR011031	78	15	52	80	73	3	0	6	8	14	0	0	0	1	7
*S-layer domains*																
S-layer family duplication domain	IPR006457	13	0	29	16	26	34	19	16	44	17	0	0	0	0	0
Sarcinarray family protein	IPR026476	0	0	6	0	4	1	0	0	2	1	0	0	0	0	0
S-layer homology domain	IPR001119	1	0	2	0	0	0	0	0	0	0	0	0	0	0	0
*Cell export and cell adhesion*																
HYR domain	IPR003410	1	5	2	0	0	0	0	0	0	0	0	0	0	0	0
CARDB domain	IPR011635	64	14	15	6	6	30	4	1	4	0	9	0	0	3	8
Collagen-binding surface protein Cna-like, B-type domain	IPR008454	4	1	2	1	8	0	6	0	2	2	0	0	0	0	0
von Willebrand factor, type A	IPR002035	55	28	17	32	14	37	23	17	42	9	28	8	0	27	7
VWA N-terminal	IPR013608	1	2	0	0	0	0	0	0	0	0	0	0	0	0	0
Adhesion lipoprotein	IPR006128	8	4	4	5	0	0	5	3	0	4	4	5	0	0	0
Adhesin B	IPR006129	0	3	4	5	0	0	3	0	0	4	0	3	0	0	0
Invasin/intimin cell-adhesion fragments	IPR008964	17	0	3	2	6	7	0	2	7	0	1	2	0	2	2
Putative cell wall binding repeat 2	IPR007253	2	2	0	0	0	0	0	0	0	0	0	0	0	0	0
Archaeosortase A	IPR014522	2	0	1	2	2	1	1	1	1	1	1	0	0	2	1
Archaeosortase B	IPR026430	0	0	0	0	0	0	0	0	0	1	0	0	0	0	0
Archaeosortase C	IPR022504	0	0	1	2	1	0	0	1	1	0	0	0	0	0	0
Exosortase/archaeosortase domain	IPR026392	5	0	2	5	3	1	1	2	2	2	1	0	1	2	1
Exosortase, EpsH	IPR013426	1	0	0	1	0	0	0	0	0	0	0	0	0	0	0
Exosortase EpsH-related	IPR019127	4	2	2	3	2	1	1	2	1	1	1	0	1	2	1
Archaeosortase family protein ArtE	IPR026485	0	0	0	0	0	0	0	0	0	0	0	0	1	0	0
Cell wall hydrolase/autolysin, catalytic	IPR002508	2	2	1	0	0	0	0	0	0	0	0	0	0	1	0
PEF-CTERM protein sorting domain	IPR017474	0	0	3	4	1	0	0	10	19	0	0	0	0	0	0
PGF-pre-PGF domain	IPR026453	1	1	18	9	8	24	0	7	21	11	0	0	0	1	1
PGF-CTERM archaeal protein-sorting signal	IPR026371	9	4	6	2	1	2	0	2	1	2	1	0	0	1	3
LPXTG cell wall anchor domain	IPR019931	3	0	1	0	0	0	1	1	0	0	0	0	0	0	0
VPXXXP-CTERM protein sorting domain	IPR026428	0	0	0	0	0	0	0	0	0	5	0	0	0	0	0
*Cellulosome-related/Dockerin*																
Cellulosome anchoring protein, cohesin domain	IPR002102	80	77	52	4	12	2	0	4	15	3	1	1	0	0	4
Dockerin domain	IPR016134	149	112	44	4	6	5	17	2	4	0	9	0	0	0	2
Dockerin type I repeat	IPR002105	1	0	5	0	0	0	0	0	0	0	0	0	0	0	1
*Carbohydrate-binding domains*																
Carbohydrate-binding domain	IPR008965	50	42	39	2	6	2	0	3	13	2	3	0	1	0	2
Carbohydrate-binding-like fold	IPR013784	14	5	3	10	19	0	0	0	0	1	3	0	0	6	1
Carbohydrate-binding, CenC-like	IPR003305	1	0	7	0	17	0	0	0	0	0	0	0	0	0	0
Carbohydrate-binding/sugar hydrolysis domain	IPR006633	27	22	5	2	2	27	3	7	8	8	0	5	2	1	8
Carbohydrate-binding domain, family 9	IPR010502	1	0	0	0	2	0	0	0	0	0	0	0	0	0	0
Bacteroidetes-associated carbohydrate-binding often N-terminal	IPR024361	0	0	1	0	0	0	0	0	0	0	0	0	0	0	0
Carbohydrate binding module, xylan-binding domain	IPR031768	0	0	0	0	1	0	0	0	0	0	0	0	0	0	0
Galactose-binding domain-like	IPR008979	12	1	17	6	39	4	4	2	1	3	0	0	0	0	0
*Menaquinones*																
3-Demethylubiquinone-9 3-methyltransferase	IPR028973	0	0	0	3	3	0	0	0	0	0	0	1	0	0	0
Succinate dehydrogenase/fumarate reductase, flavoprotein subunit	IPR014006	0	0	1	2	1	0	0	0	0	1	0	0	0	0	1
UbiE/COQ5 methyltransferase	IPR004033	3	0	0	2	2	1	0	1	0	0	0	0	0	0	0
UbiE/COQ5 methyltransferase, conserved site	IPR023576	3	0	0	2	2	0	0	0	1	0	0	0	0	0	0
NrfD family	IPR005614	3	0	0	4	3	0	0	0	0	0	0	0	0	0	3
Futalosine hydrolase	IPR019963	0	0	0	1	1	0	0	0	0	0	0	0	0	0	0
Cyclic dehypoxanthine futalosine synthase	IPR022431	1	1	0	1	1	0	0	0	0	0	0	0	0	0	1
Aminodeoxyfutalosine synthase	IPR022432	0	0	0	1	1	0	0	0	0	0	0	0	0	0	0
Menaquinone synthesis (chorismate dehydratase & naphthoate synthase)	IPR003773	8	6	0	2	2	0	0	0	0	0	0	0	0	0	2
FO synthase, subunit 2	IPR020050	3	3	1	3	3	2	1	2	1	2	2	2	2	2	3
*Phenazines*																
Phenazine biosynthesis PhzF protein	IPR003719	1	1	4	0	0	2	0	0	0	0	0	0	0	2	0

**Table 3:** Nomenclature.

*Central methanogenic pathway*	
Fmd	Formylmethanofuran (CHO-MFR) dehydrogenase
Ftr	Formylmethanofuran-tetrahydromethanopterin (H_4_MPT) formyltransferase
Mch	N^5^,N^10^-methenyl-H_4_MPT cyclohydrolase
Mtd	F_420_H_2_-dependent methylene -H_4_MPT dehydrogenase
Mer	N^5^,N^10^-methylene-H_4_MPT reductase
Mtr	N^5^-methyl-H_4_MPT:coenzyme M (CoM) methyltransferase
Mcr	Methyl coenzyme M (CH_3_-CoM) reductase
Mdh	Methanol dehydrogenase
Fae/Hps	Fusion protein of formaldehyde activating enzyme/ hexulose-6-phosphate synthase
MetF	N^5^,N^10^-methylene tetrahydrofolate (H_4_HF) reductase analogue
*Electron transport*	
Mvh	F_420_-nonreducing hydrogenase
Vho	Methanophenazine-dependent hydrogenase
Fpo	F_420_H_2_:phenazine oxidoreductase
Fqo	F_420_H_2_:quinone oxidoreductase
Hdr	Coenzyme B-coenzyme M heterodisulfide (CoB-S-S-CoM) reductase
Frh	F_420_-dependent hydrogenase
Ech	Ferredoxin-dependent hydrogenase
MePh/MePhH_2_	Methanophenazine
MQ/MQH_2_	Menaquinone
Cyt *b*	Cytochrome *b*
Cyt *c *	Cytochrome *c*
MHC	Multiheme *c*-type cytochrome
Rieske	Rieske cytochrome *b* complex
Orf7	Pseudoperiplasmic *b*-type cytochrome
Nar	Nitrate reductase
Nap	Periplasmic nitrate reductase
Nrf	Nitrite reductase
