# Supplementary material for: Reverse Methanogenesis and Respiration in Methanotrophic Archaea
Source: Archaea. 2017 Jan 5;2017:1654237. doi: 10.1155/2017/1654237 (PMC5244752; doi:10.1155/2017/1654237)
Supplement: Supplementary file 1 — This supplementary material contains data on domain based (meta)genome comparison of selected metagenomes of methanotrophs and genomes of selected archaea that belongs to the article “Reverse methanogenesis, and respiration in methanotrophic archaea” by Peer H.A. Timmers, Cornelia U. Welte, jasper J. Koehorst, Caroline M. Plugge, Mike S. M. Jetten and Alfons J.M. Stams. [file 1654237.f1.pdf]

Table S1: Domain based (meta)genome comparison of selected metagenomes of methanotrophs and genomes of selected methanogens*

	Interpro ID	ANME-1-s	ANME-1-m	ANME-2a	ANME-2d-h	ANME-2d-a	AM	A	M-1	M-2	M-3	H-1	H-2	H-3	H-4	S
**Dissimilatory sulfate reduction**																
Sulphate adenylyltransferase, small subunit	IPR011784	0	0	0	0	0	0	0	0	1	0	0	0	0	1	0
Sulphate adenylyltransferase, large subunit (Sat)	IPR011779	0	0	0	1	0	0	0	0	1	0	0	0	0	1	0
Sulphate adenylyltransferase catalytic domain (Sat)	IPR024951	1	0	0	1	1	0	0	0	0	1	0	0	0	0	1
Sulphate adenylyltransferase (Sat)	IPR002650	0	0	0	1	1	0	0	0	0	1	0	0	0	0	1
Phosphoadenosine phosphosulphate/adenosine 5'-phosphosulphate reductase	IPR004511	0	0	0	1	1	0	0	0	1	1	0	0	0	0	0
Adenosine 5'-phosphosulphate reductase	IPR011798	0	0	0	1	1	0	0	0	1	1	0	0	0	0	0
Phosphoadenosine phosphosulphate reductase	IPR002500	4	1	2	4	3	2	1	2	4	3	2	0	4	3	0
Phosphoadenosine phosphosulphate/adenosine 5'-phosphosulphate reductase	IPR004511	0	0	0	1	1	0	0	0	1	1	0	0	0	0	0
Adenylylsulphate reductase, alpha subunit (Apr)	IPR011803	0	0	0	0	0	0	0	0	0	0	0	0	0	0	1
Adenylylsulphate reductase, beta subunit (Apr)	IPR011802	1	0	0	0	0	0	0	0	0	0	0	0	0	0	1
Adenylylsulphate reductase, beta subunit, C-terminal (Apr)	IPR022738	1	0	0	0	0	0	0	0	0	0	0	0	0	0	1
Sulphite reductase, dissimilatory-type alpha subunit (Dsr)	IPR011806	0	0	0	0	0	0	0	0	0	0	0	0	0	0	1
Sulphite reductase, dissimilatory-type beta subunit (Dsr)	IPR011808	0	0	0	0	0	0	0	0	0	0	0	0	0	0	1
Dissimilatory sulphite reductase D	IPR014793	0	0	0	0	0	0	0	0	0	0	0	0	0	0	1
Sulphur relay, DsrE/F-like protein	IPR003787	4	1	3	8	9	4	2	0	1	0	0	3	3	3	3
**Assimilatory sulfate reduction**																
SLC26A/SulP transporter	IPR001902	0	1	0	0	0	0	0	0	0	0	0	1	1	0	0
ATP-sulfurylase PUA-like domain	IPR025980	0	0	0	1	1	0	0	0	0	1	0	0	0	0	1
PUA-like domain	IPR015947	30	12	12	14	13	15	11	16	20	13	13	10	13	11	15
Adenosine specific kinase	IPR007153	9	6	0	3	3	0	3	0	0	0	0	3	0	3	3
Adenylyl-sulfate kinase	IPR002891	8	1	0	1	1	0	1	0	0	0	0	0	0	2	2
Sodium/sulphate symporter, conserved site	IPR031312	0	0	1	1	2	0	1	0	0	0	1	0	0	0	0
Sodium/sulphate symporter	IPR001898	11	0	2	2	5	0	0	0	0	0	2	4	0	0	0
Nitrite/sulphite reductase iron-sulphur/sirohaem-binding site	IPR006066	9	3	5	1	8	12	5	11	6	11	5	5	0	8	1
Nitrite/sulphite reductase 4Fe-4S domain	IPR006067	7	1	3	3	4	4	2	4	2	4	2	2	0	4	3
Nitrite/Sulfite reductase ferredoxin-like domain	IPR005117	16	3	7	8	10	9	4	10	4	8	5	4	0	7	9
Thiosulphate sulfurtransferase, conserved site	IPR001307	3	3	0	1	1	0	1	0	0	2	0	1	0	0	0

Table S1 (continued): Domain based (meta)genome comparison of selected metagenomes of methanotrophs and genomes of selected methanogens*

	Interpro ID	ANME-1-s	ANME-1-m	ANME-2a	ANME-2d-h	ANME-2d-a	AM	A	M-1	M-2	M-3	H-1	H-2	H-3	H-4	S
**Heme-copper oxidase subunit II**																
Multicopper oxidase, type 1	IPR001117	0	0	0	2	2	0	0	0	0	0	0	0	0	0	0
Multicopper oxidase, type 2	IPR011706	0	0	0	2	3	0	0	0	0	0	0	0	0	0	0
Multicopper oxidase, type 3	IPR011707	0	0	0	2	1	0	0	0	0	0	0	0	0	0	0
Multicopper oxidase, copper-binding site	IPR002355	0	0	0	2	1	0	0	0	0	0	0	0	0	0	0
Multicopper oxidases, conserved site	IPR033138	0	0	0	1	1	0	0	0	0	0	0	0	0	0	0
Copper centre Cu(A), identified in cytochrome c oxidase and nitrous oxide reductase	IPR001505	0	0	0	2	2	0	0	0	0	0	0	0	0	0	2
EfeO-type cupredoxin-like domain, iron uptake system	IPR028096	0	0	0	6	7	5	0	0	0	1	0	0	0	2	1
Multi-domain cupredoxins, periplasmic domain of cytochrome c oxidase subunit II	IPR008972	2	2	6	43	45	18	0	2	6	8	0	2	2	6	4
**Nitrate reduction**																
Flavoprotein pyridine nucleotide cytochrome reductase	IPR001709	0	0	0	10	0	0	0	0	4	0	0	0	0	0	0
Rieske iron-sulphur protein, C-terminal	IPR005805	0	0	0	6	6	3	0	0	0	0	0	0	0	0	0
Rieske [2Fe-2S] iron-sulphur domain	IPR017941	3	0	0	13	6	6	0	3	12	0	0	3	0	0	0
Cytochrome c nitrite reductase, small subunit (NrfH)	IPR017571	0	0	0	2	1	0	0	0	0	0	0	0	0	0	0
NapC/NirT cytochrome c, N-terminal	IPR005126	0	1	0	3	3	0	0	0	0	0	0	0	0	0	0
Cytochrome c552 (NrfA)	IPR003321	0	0	0	3	3	0	0	1	1	0	0	0	0	0	0
Cytochrome cd1-nitrite reductase-like, haem d1 domain	IPR011048	2	1	0	2	0	2	0	1	0	0	0	0	1	0	0
DMSO/Nitrate reductase chaperone, NarJ	IPR020945	6	3	0	9	9	0	0	0	0	0	0	0	0	0	6
NarG-like domain	IPR023234	6	0	2	5	7	3	2	2	3	2	0	0	0	0	12
Periplasmic nitrate reductase NapB subunit, heme binding domain	IPR027406	0	0	0	1	0	0	0	0	1	0	0	0	0	0	0
Respiratory nitrate reductase beta, C-terminal	IPR029263	0	0	0	0	2	0	0	0	0	0	0	0	0	0	0
Nitrate reductase chaperone, NarJ	IPR003765	0	0	0	0	1	0	0	0	0	0	0	0	0	0	0
Nitrate reductase, gamma subunit	IPR003816	0	0	0	0	1	0	0	0	0	0	0	0	0	0	0
Nitrate reductase, alpha subunit	IPR006468	0	0	0	0	1	0	0	0	0	0	0	0	0	0	0
Nitrate reductase, beta subunit	IPR006547	0	0	0	0	1	0	0	0	0	0	0	0	0	0	0
DMSO/Nitrate reductase chaperone	IPR020945	6	3	0	9	9	0	0	0	0	0	0	0	0	0	6
Nitrite reductase [NAD(P)H] small subunit, NirD	IPR012748	1	0	0	0	0	0	0	0	0	0	0	0	0	0	0
CbbQ/NirQ/NorQ, C-terminal	IPR013615	2	2	0	0	0	0	0	0	1	1	0	0	0	0	0
Cytochrome c-552/DMSO reductase-like, haem-binding domain	IPR019020	0	0	0	4	6	0	0	0	0	0	0	0	0	0	0
Copper centre Cu(A), has been identified in cytochrome c oxidase and nitrous oxide reductase	IPR001505	0	0	0	2	2	0	0	0	0	0	0	0	0	0	2
Cytochrome c-552/4, tetra-heme cytochrome	IPR023155	11	2	17	22	20	2	0	6	3	16	0	0	0	0	3
Heme b synthase (heme D1)	IPR030896	0	0	1	1	1	[illegible]	0	1	1	1	0	0	0	0	0
Siroheme synthase, central domain (for nitrite reductase)	IPR028281	0	0	1	0	1	0	0	0	1	0	0	0	0	1	0
Sirohaem synthase, N-terminal (for nitrite reductase)	IPR006367	9	0	2	2	2	2	2	3	2	2	2	2	2	2	2
12,18-didecarboxysiroheme deacetylase	IPR030894	1	0	1	1	1	1	0	0	1	1	0	0	0	0	0

Table S1 (continued): Domain based (meta)genome comparison of selected metagenomes of methanotrophs and genomes of selected methanogens*

	Interpro ID	ANME-1-s	ANME-1-m	ANME-2a	ANME-2d-h	ANME-2d-a	AM	A	M-1	M-2	M-3	H-1	H-2	H-3	H-4	S
NADH:cytochrome b5 reductase (CBR)	IPR001834	0	0	4	12	0	4	0	4	4	4	0	0	0	0	0
Cytochrome b5-like heme/steroid binding domain	IPR001199	3	0	0	6	0	3	0	3	3	3	0	0	0	0	0
Cytochrome b/b6, C-terminal	IPR005798	0	0	0	2	2	0	0	0	0	0	0	0	0	0	0
Cytochrome b/b6-like domain	IPR027387	0	0	0	2	2	0	0	0	0	0	0	0	0	0	0
Cytochrome b/b6, N-terminal	IPR005797	0	0	0	1	1	0	0	0	0	0	0	0	0	0	0

* Domain abundance in every (meta)genome is indicated by numbers which are darkened according to abundance. S-AOM performing ANME include ANME-1-s [1], ANME-1-m [2], and ANME-2a [3]. N-AOM performing ANME include ANME-2d-h [4] and ANME-2d-a [5]. The acetoclastic (A) and methylotrophic (M) methanogens include *Methanosarcina acetivorans* C2A (AM), *Methanosaeta concilii* GP6 (A), *Methanococcoides burtonii* DSM6242 (M-1), *Methanolobus tindarius* DSM2278 (M-2), and *Methanohalophilus mahii* DSM5219 (M-3). Hydrogenotrophic methanogens (H) include *Methanospirillum hungatei* JF-1 (H-1), *Methanobacterium formicicum* DSM3637 (H-2), *Methanococcus maripaludis* C5 (H-3), and *Methanoregula formicica* SMSP (H-4). The sulfate-reducing archaeon (S) is *Archaeoglobus fulgidus* DSM 4304.

Table S2: Gene structure of the gene containing the Fae domain found in the metagenomes of ANME-1 [1, 2]*

Meyerdierks et al.,2010		
**Gene position**	**Interpro identifier**	
1996565-1995378	IPR013785	Aldolase-type TIM barrel
	IPR011060	Ribulose-phosphate binding barrel
	IPR014826	Formaldehyde-activating enzyme
	IPR001754	Orotidine 5'-phosphate decarboxylase domain
	IPR014826	Formaldehyde-activating enzyme
	IPR020568	Ribosomal protein S5 domain 2-type fold
	IPR014826	Formaldehyde-activating enzyme
Stokke et al.,2012		
**Gene position**	**Interpro identifier**	
18911-1996565	IPR013785	Aldolase-type TIM barrel
	IPR011060	Ribulose-phosphate binding barrel
	IPR014826	Formaldehyde-activating enzyme
	IPR001754	Orotidine 5'-phosphate decarboxylase domain
	IPR014826	Formaldehyde-activating enzyme
	IPR020568	Ribosomal protein S5 domain 2-type fold
	IPR014826	Formaldehyde-activating enzyme

*The position of the genes corresponds to the position on the contig where the specific domains were found.

Table S3: Gene structure of the iron hydrogenase gene found in the metagenomes of ANME-1 (gene 1) and the surrounding genes (gene 2 and gene 3)*

Meyerdierks et al., 2010	**Gene position**	**Interpro identifier**	
**Gene 1**	925906-927615	IPR003149	Iron hydrogenase, small subunit
		IPR019574	NADH:ubiquinone oxidoreductase, subunit G, iron-sulphur binding
		IPR009016	Iron hydrogenase
		IPR013352	Iron hydrogenase, subset
		IPR001041	2Fe-2S ferredoxin-type iron-sulfur binding domain
		IPR017896	4Fe-4S ferredoxin-type, iron-sulphur binding domain
		IPR000283	NADH:ubiquinone oxidoreductase, 75kDa subunit, conserved site
		IPR003149	Iron hydrogenase, small subunit
		IPR004108	Iron hydrogenase, large subunit, C-terminal
		IPR019574	NADH:ubiquinone oxidoreductase, subunit G, iron-sulphur binding
**Gene 2**	927628-928404	IPR011538	NADH-ubiquinone oxidoreductase 51 kDa subunit, FMN-binding domain
		IPR019554	Soluble ligand binding domain
		IPR019575	NADH-ubiquinone oxidoreductase 51 kDa subunit, iron-sulphur binding domain
		IPR001949	NADH:ubiquinone oxidoreductase, 51kDa subunit, conserved site
		IPR019575	NADH-ubiquinone oxidoreductase 51 kDa subunit, iron-sulphur binding domain
**Gene 3**	928501-928854	IPR011538	NADH-ubiquinone oxidoreductase 51 kDa subunit, FMN-binding domain
		IPR002023	NADH-quinone oxidoreductase subunit E-like
		IPR012336	Thioredoxin-like fold

* The position of the genes corresponds to the position on the contig where the specific domains were found.

Table S3 (continued): Gene structure of the iron hydrogenase gene found in the metagenomes of ANME-1 (gene 1) and the surrounding genes (gene 2 and gene 3)*

Stokke et al., 2012	**Gene position**	**Interpro identifier**	
**Gene 1**	927615-925906	IPR003149	Iron hydrogenase small subunit
		IPR019574	NADH:ubiquinone oxidoreductase, subunit G, iron-sulphur binding
		IPR009016	Iron hydrogenase
		IPR013352	Iron hydrogenase, subset
		IPR001041	2Fe-2S ferredoxin-type iron-sulfur binding domain
		IPR017896	4Fe-4S ferredoxin-type, iron-sulphur binding domain
		IPR017900	4Fe-4S ferredoxin, iron-sulphur binding, conserved site
		IPR012675	Beta-grasp domain
		IPR000283	NADH:ubiquinone oxidoreductase, 75kDa subunit, conserved site
		IPR003149	Iron hydrogenase, small subunit
		IPR004108	Iron hydrogenase, large subunit, C-terminal
**Gene 2**	928404-927628	IPR011538	NADH-ubiquinone oxidoreductase 51 kDa subunit, FMN-binding domain
		IPR019554	Soluble ligand binding domain
		IPR019575	NADH-ubiquinone oxidoreductase 51 kDa subunit, iron-sulphur binding domain
		IPR001949	NADH:ubiquinone oxidoreductase, 51kDa subunit, conserved site
**Gene 3**	928854-928501	IPR011538	NADH-ubiquinone oxidoreductase 51 kDa subunit, FMN-binding domain
		IPR002023	NADH-quinone oxidoreductase subunit E-like
		IPR002023	NADH-quinone oxidoreductase subunit E-like
		IPR012336	Thioredoxin-like fold

*The position of the genes corresponds to the position on the contig where the specific domains were found.

References

1. Stokke, R., et al., *Integrated metagenomic and metaproteomic analyses of an ANME-1-dominated community in marine cold seep sediments.* Environ Microbiol, 2012. **14**(5): p. 1333-1346.
2. Meyerdierks, A., et al., *Metagenome and mRNA expression analyses of anaerobic methanotrophic archaea of the ANME-1 group.* Environ Microbiol, 2010. **12**(2): p. 422-439.
3. Wang, F.P., et al., *Methanotrophic archaea possessing diverging methane-oxidizing and electron-transporting pathways.* ISME J, 2014. **8**(5): p. 1069-1078.
4. Haroon, M.F., et al., *Anaerobic oxidation of methane coupled to nitrate reduction in a novel archaeal lineage.* Nature, 2013. **500**(7468): p. 567-570.
5. Arshad, A., et al., *A metagenomics-based metabolic model of nitrate-dependent anaerobic oxidation of methane by Methanoperedens-like archaea.* Front Microbiol, 2015. **6**: p. 1423, doi: 10.3389/fmicb.2015.01423.
